# Interacting ultracold atomic kicked rotors: loss of dynamical localization

**DOI:** 10.1038/srep41139

**Published:** 2017-01-24

**Authors:** Pinquan Qin, Alexei Andreanov, Hee Chul Park, Sergej Flach

**Affiliations:** 1 Center for Theoretical Physics of Complex Systems, Institute for Basic Science, Daejeon, South Korea; 2 New Zealand Institute for Advanced Study, Center for Theoretical Chemistry & Physics, Massey University, Auckland, New Zealand

## Abstract

We study the fate of dynamical localization of two quantum kicked rotors with contact interaction, which relates to experimental realizations of the rotors with ultra-cold atomic gases. A single kicked rotor is known to exhibit dynamical localization, which takes place in momentum space. The contact interaction affects the evolution of the relative momentum *k* of a pair of interacting rotors in a non-analytic way. Consequently the evolution operator *U* is exciting large relative momenta with amplitudes which decay only as a power law 1/*k*^4^. This is in contrast to the center-of-mass momentum *K* for which the amplitudes excited by *U* decay superexponentially fast with *K*. Therefore dynamical localization is preserved for the center-of-mass momentum, but destroyed for the relative momentum for any nonzero strength of interaction.

The quantum kicked rotor (QKR) model is a canonical model to explore quantum chaos^1^,^2^. It describes a quantum rotor degree of freedom which is periodically kicked by a force periodic in the angle. The QKR enjoys dynamical localization (DL) - i.e. the arresting of the growth of the momentum despite the absence of a cutoff in the frequency of the kick drive. DL was first discovered numerically by Casati, Chirikov, Ford, and Izrailev^3^ and later confirmed experimentally for Rydberg atoms in a microwave field^4^,^5^ and ultracold atomic gases in a modulated standing wave of a near-resonant laser^6^. A recent work reports on the experimental observation of DL with laser-kicked molecular rotors^7^. Coupled kicked rotors can also be realized by placing atoms in pulsed, incommensurate optical lattices^8^. If the driving period is an irrational multiple of 2*π*, the rotor is localized in the momentum space, even though the classical counterpart shows diffusive momentum growth. This happens because classical chaotic diffusion is suppressed by quantum interference effects. The mechanism of DL was described in a seminal paper by Fishman, Grempel and Prange^9^. These authors demonstrated that the kicked rotor model maps directly to an Anderson-like model with a quasi-periodic potential, which originates from the irrational driving periods. Therefore DL is closely related to Anderson localization of waves in truly random (uncorrelated) potentials.

The original quantum kicked rotor corresponds to a single quantum particle problem. The effect of interactions on Anderson localization has been attracting a lot of interest recently and several theoretical studies considered various versions of interacting kicked rotors. In ref. 10 a similar problem was studied for a simpler, integrable model of linear rotors^9^, where localization can survive in the presence of interactions due to integrability. The authors of ref. 11 analyzed coupled relativistic rotors which might be applicable to fermions in pulsed magnetic fields, and reported that DL can be destroyed by suitable parameter tuning. In ref. 12, two kicked rotors with product sinusoidal interaction at the kick were studied with respect to temporal fluctuations in the reduced density matrix. In ref. 13, the coupling was sinusoidal depending on the two rotors relative coordinates: recovering of the chaotic behavior was found above some kicking threshold in the semi-classical approximation. In ref. 14, the interaction at the kick of the kicked rotors contained both product and relative coordinate dependent sinusoidal terms. Localization was found for weak coupling and quasi-diffusive regime was found for stronger interaction with a complex intermediate regime.

From the experimental perspective, interaction between rotors is negligible for Rydberg atoms and laser-kicked molecular rotors. However the interaction between ultracold atoms in a Bose-Einstein condensate (BEC) can be substantial, and even tunable using Feshbach resonances^15^, which is particularly true for sodium atoms used in ref. 6. The atom-atom interaction in this case is typically of a contact type, i.e. the atoms interact through a *δ*(*x*_1_ − *x*_2_) potential^15^. For the experimental realization in ref. 6, this interaction of BEC atoms persists at all times - in contrast to the kick potential, and in contrast to the theoretical studies discussed above, which consider a kicked (time-dependent) interaction. A *δ*(*x*) interaction is long-ranged in momentum space, and can therefore have a qualitatively strong impact on DL for interacting ultracold atoms. Will DL survive, or not?

In this work, we address this question. We consider two bosons interacting via a *δ*-function potential that are driven by a periodic kicking potential. The wave function for two *δ*-function interacting bosons is computed. We use the center of mass and relative coordinates with appropriate periodic boundary conditions. A repulsive *δ*-interaction is considered, that does not lead to the appearance of a bound state. In the chosen basis the matrix elements of the time evolution operator show different decay rates along the center of mass (superexponential) and the relative momentum (algebraic) directions. Due to this qualitative difference in the decay properties of the matrix elements, dynamical localization (i.e. exponential localization with a finite localization length) is destroyed for the relative momentum, while being preserved for the center-of-mass momentum.

## Model and Methods

We consider two bosons moving on a ring [0, 2*π*) with *δ*-function interaction and periodic kicking potential. The Hamiltonian of the model is given by:


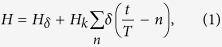


where 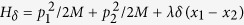 is the two-body Hamiltonian of the Lieb-Liniger model^16^, 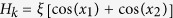, *λ* is the interaction strength, *ξ* is the kicking strength. In the experimental setup of ref. 6, the kicking potential, a standing wave of a near resonant light, is cigar-shape and not on a ring. That will in general change the boundary conditions, and momentum selection rules, as compared to a ring structure with periodic boundary conditions. These changes are however not of qualitative nature for non-interacting particles, while multiple collisions for interacting particles could yield additional sensitivity to boundary conditions. However, our main results will be obtained from analyzing the impact of only *one* kick. Furthermore, recent toroidal Bose-Einstein condensates have been experimentally realized which emulate periodic boundary conditions^17^. If DL is destroyed in just a two-body interacting model, we expect it also be destroyed in the experimental many-body interaction case. Therefore we can view our model as a first step towards the consideration of a many-body interacting system and a simple paradigmatic case of just two interacting atoms which is the building block of reaching out to many-body interactions.

We start by computing the wave function of the two bosons system with *δ*-function interaction. It can be represented in a center of mass and relative coordinates frame - (*y*_1_, *y*_2_), where *y*_1_ = *x*_1_ + *x*_2_, *y*_2_ = *x*_1_ − *x*_2_. In this frame, the first part of (1) reads


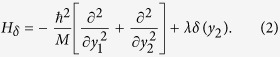


It splits in two parts, 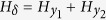, where 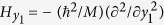, 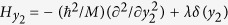. 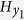 describes a free moving particle and 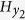 describes a single particle with *δ*-function potential. The wave function of the complete system is 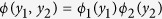 - the product of two single particle wave functions *ϕ*_1_ and *ϕ*_2_, that satisfy 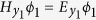, 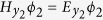. The total eigenenergy of the system is 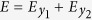. Because of the periodicity, the complete wave function satisfies 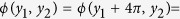

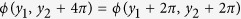
. This can be simplified into three identities: *ϕ*_1_(*y*_1_) = *ϕ*_1_(*y*_1_ + 4*π*), 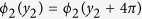, 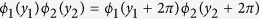, which serve as the periodic boundary conditions for *ϕ*_1_ and *ϕ*_2_.

The wavefunction for the free moving particle is:


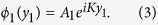


The periodic boundary conditions 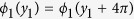 select the quantized values of *K: e*^*i*4*Kπ*^ = 1, giving *K* = 0, ±1/2, ±1, ±3/2, … The normalization condition yields 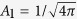, and the eigenenergy 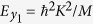.


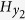
 describes a massive particle on a one-dimensional ring of circumference 4*π* and a *δ*-function singularity at *y*_2_ = 0. The eigenstates of this problem can be either symmetric or antisymmetric around *y*_2_ = 0. Since rotors are bosons, the wave function should be invariant under permutation *x*_1_↔*x*_2_ and only symmetric functions *ϕ*_2_(*y*_2_) = *ϕ*_2_(−*y*_2_) are allowed. Then the derivative is an antisymmetric function 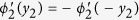. The wave function is continuous at 0: *ϕ*_2_(+0) = *ϕ*_2_(−0), but its derivative has a jump: 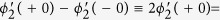

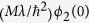
. From the periodic boundary condition 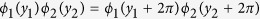, it follows that *ϕ*_2_(*y*_2_) = *e*^*i*2*Kπ*^*ϕ*_2_(*y*_2_ + 2*π*) and *ϕ*_2_(*y*_2_) = *ϕ*_2_(*y*_2_ + 4*π*). It is worth noting that the center of mass and relative momenta do not decouple completely, due to the boundary conditions.

With these boundary conditions it is easy to compute the wave function *ϕ*_2_ (see Supplementary material for more details):


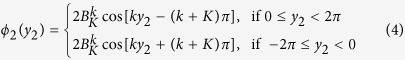



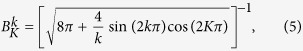



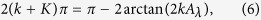


with *A*_*λ*_ = *ħ*^2^/*Mλ* being a dimensionless inverse interaction strength and the eigenenergy 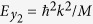. We use *A*_*λ*_ to measure the strength of the interaction, to which it is inversely proportional. We attach the momenta *K* and *k* to the full wavefunction - 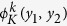. Now the eigenstates of two bosons with *δ*-function interaction can be written as 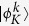, the corresponding wave function is


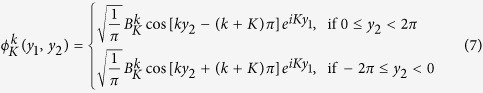


The eigenenergy is given by 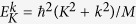. The wavefunctions are symmetric with respect to *k*: if *k* is a solution of Eq. (6) then −*k* is also a solution. As follows from Eq. (4), the wave function with *k* and −*k* are exactly the same for integer *K* or differ by a global phase *π* for half integer *K*. Consequently 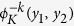 is equivalent to 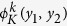, reflecting the bosonic nature of the rotors. In the following discussion, we only consider the *k* > 0 case.

In the presence of a periodic driving potential in Eq. (1), the dynamics is described by the time evolution operator over one period *U* (Floquet propagator)^18^. The matrix of *U* is computed below in the basis of 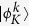 (see also the Supplementary material). Starting from some initial state |*ψ*(0)〉 of the rotors, the final state |*ψ*(*NT*)〉 after *N* driving periods is obtained the initial state by matrix multiplication:


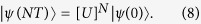


## Results and Discussion

For periodically kicked interacting rotors, the Floquet operator reads


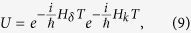


where *H*_*δ*_ is given by Eq. (2) and *H*_*k*_ = 2*ξ* cos(*y*_1_/2) cos(*y*_2_/2). In the basis of 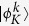, the matrix elements of *U* become


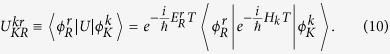


As shown in the Supplementary material, for 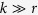 and fixed *K, R* this matrix element scales as


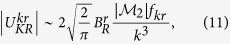



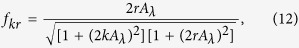


where 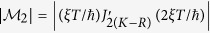. For a fixed *A*_*λ*_ and large enough *k* and *r* (such that 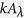, 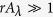), the matrix elements of *U* decay as


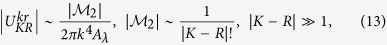


with the asymptotics along the center-of-mass momentum direction following from the asymptotic properties of Bessel functions^19^ (Notice that the dependence on |*K* − *R*| is through the index of the Bessel function). Therefore, for large *k* and *r*, the matrix element 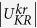 decays super-exponentially fast with the center of mass *K* momentum (i.e. faster than exponential and asymptotically as a factorial), due to the scaling of 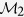 which is controlled by the first order derivative of the Bessel function (13) (the same conclusion holds for any values of *r* and *k*, see Supplementary material). The decay along the relative momentum *k* direction however is a power law *k*^−4^, reflecting the presence of a singular *δ*-function interaction. This is our key result: super-exponential decay of the matrix element ensures the survival of dynamical localization for the center-of-mass momentum, while the power-law decay destroys it for the relative momentum (in the sense of exponential localization with a finite localization length). Smearing the *δ*-function of the interaction into a smooth (analytic) one will introduce an energy (wave number) cutoff *k*_max_ and corresponding exponential matrix element decay for energies beyond that cutoff, ensuring survival of the DL for the relative momenta in this case. This follows from the observation that the matrix element is essentially a Fourier transform of a function related to the interaction potential with respect to *k* (see Supplementary material). It is non-analytic in the case of the *δ*-interaction (since its first derivative has a jump), while it is analytic for smooth (analytic) interaction potentials. The asymptotic convergence of a Fourier transform is exponential for analytic functions, but only a power law for the non-analytic ones.

**Figure 1:**
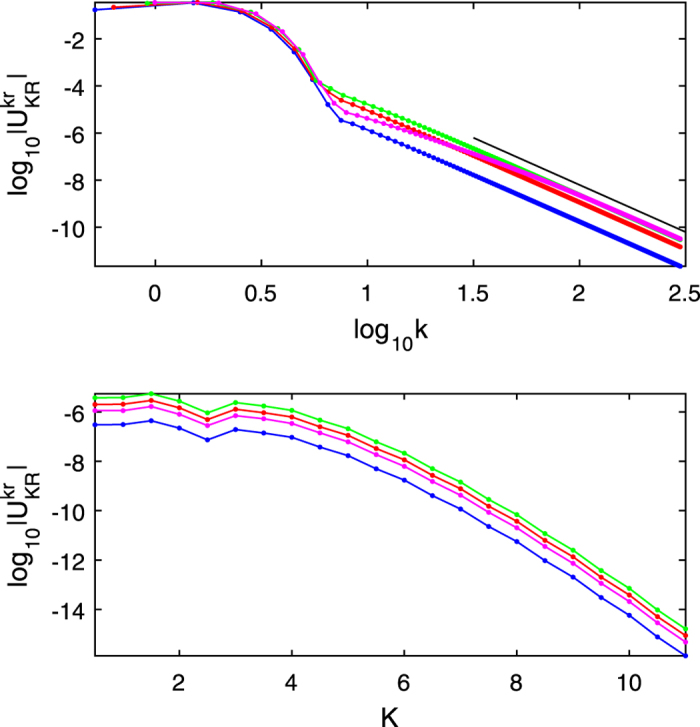
Matrix element (10) plotted vs. center-of-mass *K* and relative *k* momenta, kicking strength *ξ* = 3 and driving period *T* = 1. The colors correspond to different inverse interactions strengths *λ*: blue - *A*_*λ*_ = 10, red - *A*_*λ*_ = 1, green - *A*_*λ*_ = 0.1, magenta - *A*_*λ*_ = 0.01. *Top*: 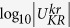 vs. log_10_ *k* with *K* = *R* = 0 and fixed *r* - the same values as for the bottom figure. The black line is a plot of −4log_10_ *k* − 0.2 and placed here for comparison. The power law decay of the matrix element is clearly observed for large values of relative momentum. *Bottom*: Plot of the matrix element 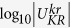 vs. *K* with *R* = 0, fixed values of relative momenta: blue -*k* = 14.001, *r* = 0.123; red -*k* = 14.011, *r* = 0.319; green -*k* = 14.108, *r* = 0.470; magenta -*k* = 14.410, *r* = 0.496. The plots curve away down from the straight line at large values of *K*, indicating a super-exponential decay.

The comparison of the numerical results to the asymptotic behavior is presented in Fig. 1. The top figure shows the decay of matrix element 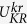 with relative momentum *k* for several values of the coupling *A*_*λ*_ indicated by colors. The other momenta *r, K, R* are fixed. The power law fit 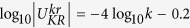 (the black line) agrees well with the numerical values of the matrix elements 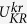 with a given *K, R, r* and 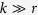. The small-*k* dependence is not sensitive to the inverse interaction strength *A*_*λ*_. The power-law decay of the matrix elements is not monotonic with *A*_*λ*_: initially (blue, red and green curves) the prefactor is decreasing, however upon further decrease of *A*_*λ*_ (magenta curve) it starts to increase and there is a non-monotonic intermediate part. This non-monotonicity can be explained from Eqs (11 and 12): for very small and very large *A*_*λ*_, *f*_*kr*_ is small, behaving as 2*rA*_*λ*_ and 1/(2*kA*_*λ*_) respectively. Therefore, with decreasing *A*_*λ*_, and for given *k, K, r, R*, the matrix element of *U* (11) will first increase from a small value and then decrease back, which is precisely the non-monotonicity observed. For small *A*_*λ*_ the power-laws have the same prefactors: this follows from (12) - for a fixed *r* and 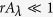, 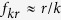 is independent of *A*_*λ*_. This is what we observe for the smallest values of *A*_*λ*_ in Fig. 1.

The bottom plot in Fig. 1 shows the decay of the matrix elements as a function of *K*: a faster than exponential decay is observed, agreeing with the asymptotic behavior (13). The prefactor also shows non-monotonicity - the matrix elements initially increase with increasing *A*_*λ*_ and later decrease - and has the same origin as above.

The impact of the decay properties of the matrix elements of *U* is observed in the evolution of an initial state 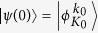 with fixed momenta *K*_0_ and *k*_0_ according to Eq. (8). This choice of initial state is well-suited to detect delocalization in the momentum space. The final state after *N* driving periods is 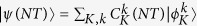, where the 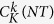 are the expansion coefficients over the eigenstates of the two-body Lieb-Liniger problem.

**Figure 2:**
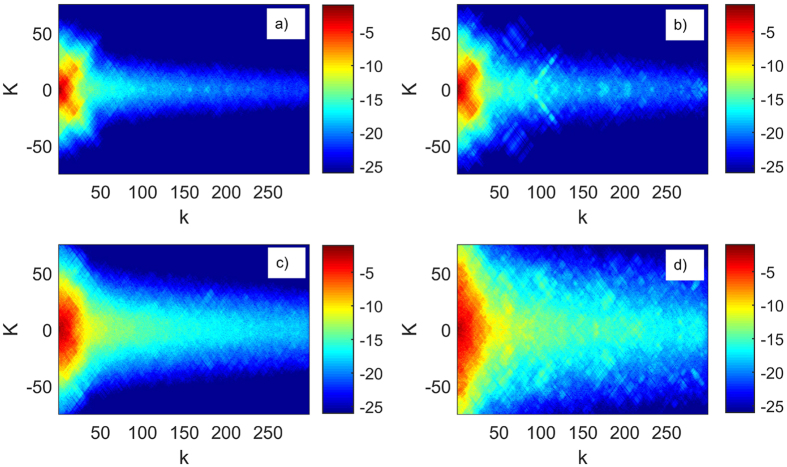
*Top*: Expansion coefficients 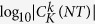 vs. momenta *K, k* for dimensionless inverse interaction strength *A*_*λ*_ = 10 and initial relative momentum *k*_0_ = 0.12. *Bottom*: 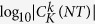 vs. *K, k*, inverse interaction strength *A*_*λ*_ = 0.1, initial relative momentum *k*_0_ = 0.47. Number of driving periods: (**a**,**c**) *N* = 100, (**b**,**d**) *N* = 5000. Parameters: kicking strength *ξ* = 3, driving period *T* = 1 and momenta cutoffs *K*_max_ = 301, *k*_max_ = 300 and *K*_0_ = 0. For both interaction values *A*_*λ*_ the extent of the wavefunction in the *C* space is growing with the number of driving periods. An increase of the interaction strength *λ (A*_*λ*_ ~ 1/*λ*) facilitates the delocalization.

Figure 2 shows the final state after *N* = 100 - left column - and *N* = 5000 - right column - driving periods for two different values *A*_*λ*_, corresponding to moderately strong interaction between the rotors. The final state gets extended along the relative momentum *k* direction. Also the extension is more pronounced with decreasing *A*_*λ*_.

**Figure 3:**
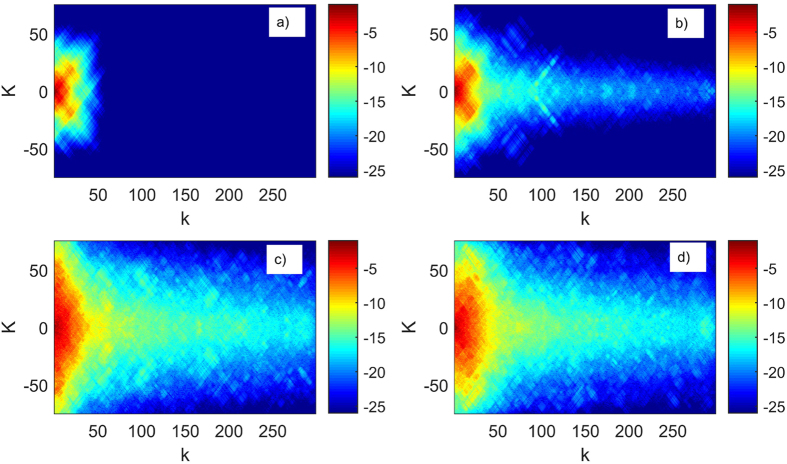
Expansion coefficients 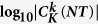 vs. *K, k* with momenta cutoffs *K*_max_ = 301, *k*_max_ = 300, kicking strength *ξ* = 3, driving period *T* = 1 and number of driving periods *N* = 5000. The inverse interaction strength decreases from (**a**–**d**) as *A*_*λ*_ = 10^14^, *A*_*λ*_ = 10, *A*_*λ*_ = 0.1, *A*_*λ*_ = 0.01 respectively. Similar to Fig. 2, the extent of the wavefunction is increasing with increasing interaction *λ*.

Figure 3 shows the amplitude distribution of the final state after *N* = 5000 driving periods for several values of *A*_*λ*_. Figure 3(a) shows the final state for the case of two essentially non-interacting rotors (*A*_*λ*_ = 10^14^). The final state is localized in both *K* and *k* momenta directions, displaying the dynamical localization of the non-interacting kicked rotor model. As the strength of the interaction is increasing, Fig. 3(b,c), the final state starts to extend along the *k* direction. The interaction between the two rotors delocalizes the state in the relative momentum *k* direction. The localization length along the center of mass momentum *K* direction also increases, as seen in Fig. 3(b,c). Figure 3(d) shows the final state for strong interaction: compared with the (c) case, the extension of the final state along the *K* direction has shrunk in a small momentum *k* region. For very large momenta *k* the amplitudes of the final state have values similar to the (c) case, since the matrix elements of *U* become independent of *A*_*λ*_ as we have discussed earlier.

In order to quantify the spreading of the initial state with *N*, we compute the evolution of the variance of the momenta:


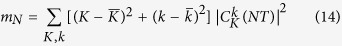



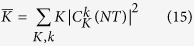



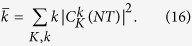


**Figure 4:**
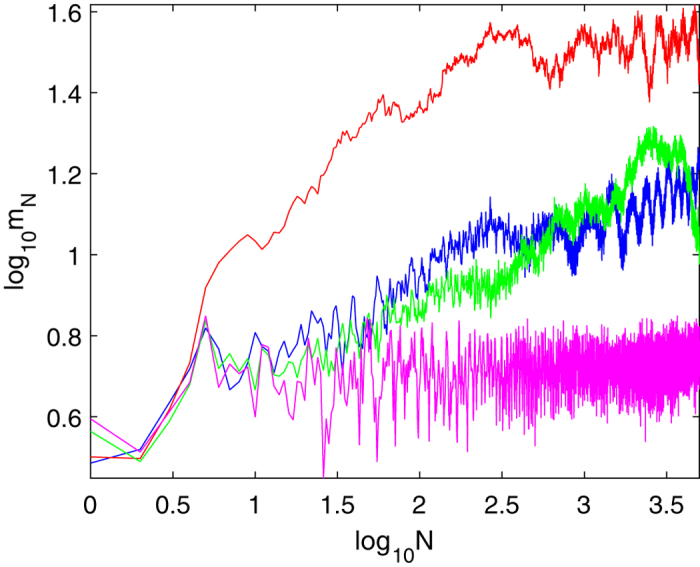
The variance of the momenta (Eq. (14)) log_10_*m*_*N*_ vs. log_10_ *N* with momenta cutoff *K*_max_ = 301, *k*_max_ = 300, kicking strength *ξ* = 3, driving period *T* = 1 and for several values of the inverse interaction strength: blue line - *A*_*λ*_ = 0.01, red line - *A*_*λ*_ = 0.1, green line - *A*_*λ*_ = 1, magenta line - *A*_*λ*_ = 10. As the interaction increases, the variance increases as well, in accord with the loss of localization along the relative momentum direction. The non-monotonic behavior at large interactions is explained in the main text.

Figure 4 shows the evolution of the variance with *N* for several strengths of the interaction *A*_*λ*_. For all the values of the interaction except *A*_*λ*_ = 10 the variance has a clearly increasing trend spanning several orders of magnitude in *N*, therefore signaling delocalization along the relative momentum *k* direction. The non-monotonic dependence of the variance on *A*_*λ*_ has the same origin as the non-monotonic dependence of the matrix elements of *U* that we discussed above.

## Conclusions

In conclusion, we studied dynamical properties of two interacting kicked quantum rotors. Due to the non-analyticity of the *δ*-function interaction, the matrix elements of the time evolution operator exhibit different decay behaviors in center-of-mass and relative momentum directions. Along the center of mass momentum direction, matrix elements decay super-exponentially. Along the relative momentum direction, matrix elements decay as a power-law with the exponent 4. As a result the center-of-mass motion remains localized like in the non-interacting case, while the relative motion becomes extended. The destruction of dynamical localization should be observable in toroidal traps^17^ and setups similar to the one used in ref. 6 using Feshbach resonances. Furthermore, while contact interaction is a good approximations, our conclusions hold for other regularized (analytic) longer ranged interactions as well, at least up to the corresponding energy cutoff which will separate the convergence criteria of the Fourier series of the contact interaction potential from its regularized version in momentum space. The extension to many interacting particles is also an interesting path. To analyze this, we need to consider many interacting atoms and study the highly complex case of many-body interactions for quantum kicked rotors. This is a challenging task, as can be observed from the complexity of results on mean field treatments in refs 20, 21, 22 which range from complete destruction of dynamical localization to modifications of Anderson transitions. However, we strongly believe, that in the many-body version of our model, this mechanism still holds, leading to the destruction of DL, and is even more efficient due to the increased number of relaxation channels.

## Additional Information

**How to cite this article**: Qin, P. *et al*. Interacting ultracold atomic kicked rotors: loss of dynamical localization. *Sci. Rep.* **7**, 41139; doi: 10.1038/srep41139 (2017).

**Publisher's note:** Springer Nature remains neutral with regard to jurisdictional claims in published maps and institutional affiliations.

## Supplementary Material

Supplementary Information

## References

[b1] Chirikov B. V. A universal instability of many-dimensional oscillator systems. Phys. Rep. 52, 263–379 (1979).

[b2] Izrailev F. M. Simple models of quantum chaos: Spectrum and eigenfunctions. Phys. Rep. 196, 299–392 (1990).

[b3] Casati G., Chirikov B. V., Izraelev F. M. & Ford J. Stochastic Behavior in Classical and Quantum Hamiltonian Systems: Volta Memorial Conference, Como, 1977 (Springer Berlin Heidelberg, Berlin, Heidelberg, 1979).

[b4] Galvez E. J., Sauer B. E., Moorman L., Koch P. M. & Richards D. Microwave ionization of h atoms: Breakdown of classical dynamics for high frequencies. Phys. Rev. Lett. 61, 2011–2014 (1988). 10038960 10.1103/PhysRevLett.61.2011

[b5] Bayfield J. E., Casati G., Guarneri I. & Sokol D. W. Localization of classically chaotic diffusion for hydrogen atoms in microwave fields. Phys. Rev. Lett. 63, 364–367 (1989). 10041054 10.1103/PhysRevLett.63.364

[b6] Moore F. L., Robinson J. C., Bharucha C., Williams P. E. & Raizen M. G. Observation of dynamical localization in atomic momentum transfer: A new testing ground for quantum chaos. Phys. Rev. Lett. 73, 2974–2977 (1994). 10057250 10.1103/PhysRevLett.73.2974

[b7] Bitter M. & Milner V. Experimental observation of anderson localization in laser-kicked molecular rotors. arXiv:1603.06918 (2016). 10.1103/PhysRevLett.117.144104 27740833

[b8] Gadway B., Reeves J., Krinner L. & Schneble D. Evidence for a quantum-to-classical transition in a pair of coupled quantum rotors. Phys. Rev. Lett. 110, 190401 (2013). 23705694 10.1103/PhysRevLett.110.190401

[b9] Fishman S., Grempel D. R. & Prange R. E. Chaos, quantum recurrences, and Anderson localization. Phys. Rev. Lett. 49, 509–512 (1982).

[b10] Keser A. C., Ganeshan S., Refael G. & Galitski V. Dynamical many-body localization in an integrable model. arXiv:1506.05455 (2015).

[b11] Rozenbaum E. B. & Galitski V. Dynamical localization of coupled relativistic kicked rotors. arXiv:1602.04425 (2016).

[b12] Nag S., Ghosh G. & Lahiri A. Quantum chaos: Reduced density matrix fluctuations in coupled systems. Physica D 204, 110–121 (2005).

[b13] Adachi S., Toda M. & Ikeda K. Quantum-classical correspondence in many-dimensional quantum chaos. Phys. Rev. Lett. 61, 659–661 (1988). 10039397 10.1103/PhysRevLett.61.659

[b14] Toloui B. & Ballentine L. E. Quantum localization for two coupled kicked rotors. arXiv:0903.4632 (2009).

[b15] Cheng C., Rudolf G., Paul J. & Eite T. Feshbach resonances in ultracold gases. Rev. Mod. Phys. 823, 1225 (2010).

[b16] Lieb E. H. & Liniger W. Exact analysis of an interacting bose gas. i. the general solution and the ground state. Phys. Rev. 130, 1605–1616 (1963).

[b17] Ramanathan A. . Superflow in a Toroidal Bose-Einstein Condensate: An Atom Circuit with a Tunable Weak Link. Phys. Rev. Lett. 106, 130401 (2011). 21517360 10.1103/PhysRevLett.106.130401

[b18] Grifoni M. & Hänggi P. Driven quantum tunneling. Phys. Rep. 304, 229–354 (1998).

[b19] Abramowitz M. & Stegun I. A. Handbook of Mathematical Functions With Formulas, Graphs, and Mathematical Tables, 1972 (Washington DC, USA; New York, USA 1972).

[b20] Shepelyansky D. L. Delocalization of quantum chaos by weak nonlinearity. Phys. Rev. Lett. 70, 1787–1790 (1993). 10053386 10.1103/PhysRevLett.70.1787

[b21] Gligoric G., Bodyfelt J. D. & Flach S. Interactions destroy dynamical localization with strong and weak chaos. EPL 96, 30004 (2011).

[b22] Cherroret N., Vermersch B., Garreau J. C. & Delande D. How Nonlinear Interactions Challenge the Three-Dimensional Anderson Transition. Phys. Rev. Lett. 112, 170603 (2014). 24836228 10.1103/PhysRevLett.112.170603

