# Supplementary material for: Interacting ultracold atomic kicked rotors: loss of dynamical localization
Source: Sci Rep. 2017 Jan 24;7:41139. doi: 10.1038/srep41139 (PMC5259732; doi:10.1038/srep41139)
Supplement: Supplementary Information [file srep41139-s1.pdf]

Supplementary information on
Interacting ultracold atomic kicked rotors: loss of dynamical localization

Pinquan Qin,[1] Alexei Andreanov,[1] Hee Chul Park,[1] and Sergej Flach[1,2]

[1] *Center for Theoretical Physics of Complex Systems, Institute for Basic Science, Daejeon, South Korea*

[2] *New Zealand Institute for Advanced Study, Center for Theoretical Chemistry & Physics, Massey University, Auckland, New Zealand*

I. THE WAVE FUNCTION OF SINGLE PARTICLE WITH δ-FUNCTION POTENTIAL AND PERIODIC BOUNDARY CONDITION

The wave function of single particle with δ-function potential can be defined as,

$$\phi_2(y_2) = \begin{cases} Ae^{iky_2} + Be^{-iky_2}, & \text{if} \quad 0 \le y_2 < 2\pi \\ Ce^{iky_2} + De^{-iky_2}, & \text{if} \quad -2\pi \le y_2 < 0 \end{cases} . \tag{1}$$

From the symmetry condition of the wave function $\phi_2(y_2) = \phi_2(-y_2)$, it follows that $C = B$ and $D = A$. Now,

$$\phi_2(y_2) = \begin{cases} Ae^{iky_2} + Be^{-iky_2}, & \text{if} \quad 0 \le y_2 < 2\pi \\ Be^{iky_2} + Ae^{-iky_2}, & \text{if} \quad -2\pi \le y_2 < 0 \end{cases} . \tag{2}$$

The condition for the derivative of the wave function $\phi_2' = -\phi_2'(-y_2)$ is automatically satisfied as requested from this equation. For the condition $\phi_2(+0) = \phi_2(-0)$, it can be satisfied when $\phi_2(0) = A + B$. Next, the discontinuity condition in the derivative $\phi_2'(+0) - \phi_2'(-0) = \left(M\lambda/\hbar^2\right)\phi_2(0)$ tell us

$$2ik(A - B) = \frac{M\lambda}{\hbar^2}(A + B) . \tag{3}$$

This equation gives,

$$A\left(1 - \frac{M\lambda}{2ik\hbar^2}\right) = B\left(1 + \frac{M\lambda}{2ik\hbar^2}\right) . \tag{4}$$

The quantization condition $\phi_1(y_1)\phi_2(y_2) = \phi_1(y_1 + 2\pi)\phi_2(y_2 + 2\pi)$, leads to $B = A\mathrm{e}^{2ik\pi}e^{2iK\pi}$. Substituting this equation into Eq.(4), we have

$$\mathrm{e}^{2i(k+K)\pi} = \frac{1 - \frac{M\lambda}{2ik\hbar^2}}{1 + \frac{M\lambda}{2ik\hbar^2}} = -\frac{\frac{M\lambda}{2\hbar^2} - ik}{\frac{M\lambda}{2\hbar^2} + ik} . \tag{5}$$

Because of

$$-e^{i2(k+K)\pi} = \frac{1 - i\tan\left(\frac{-2(k+K)\pi+\pi}{2}\right)}{1 + i\tan\left(\frac{-2(k+K)\pi+\pi}{2}\right)} , \tag{6}$$

win find finally the following equation on k:

$$2(k + K)\pi = \pi - 2\arctan\left(2kA_\lambda\right) , \tag{7}$$

where $A_\lambda = \hbar^2/M\lambda$.

Next we compute the normalization of the wavefunction:

$$\int_0^{2\pi} |\phi_2|^2 dy_2 = 1/2 . \tag{8}$$

Substituting the wavefunction (2) into this equation and carrying out the integration, we find,

$$|A|^{-1} = \sqrt{8\pi + \frac{4}{k}\sin(2k\pi)\cos(2K\pi)} . \tag{9}$$

The phase ϕ of A is unfixed, because it is a global phase of the wave function and can take any value. We fix ϕ by requiring the wave function ϕ_2 to be real. Therefore $A = B^*$. Considering $A = |A|e^{i\varphi}$ and $B = Ae^{2ik\pi}e^{2iK\pi}$, we find,

$$\varphi = -(k+K)\pi + 2\pi m \ , \ m = 0, \pm 1, \pm 2, ... \tag{10}$$

Here we only consider $\varphi = -(k+K)\pi$. To wrap it up, the wave function of single particle with δ function potential is solved as:

$$\phi_2(y_2) = \begin{cases} 2B_K^k \cos[ky_2 - (k+K)\pi], & \text{if} \quad 0 \le y_2 < 2\pi \\ 2B_K^k \cos[ky_2 + (k+K)\pi], & \text{if} \quad -2\pi \le y_2 < 0 \end{cases} , \tag{11}$$

where

$$B_K^k = \left[\sqrt{8\pi + \frac{4}{k}\sin(2k\pi)\cos(2K\pi)}\right]^{-1} \tag{12}$$

$$2(k+K)\pi = \pi - 2\arctan(2kA_\lambda) \ . \tag{13}$$

II. THE DECAY BEHAVIOR OF THE ELEMENTS OF THE U MATRIX

Here we present the detailed derivation of the decay of the elements of the Floquet matrix U as a function of center-of-mass and relative momenta.

The matrix element of U is given by

$$U_{KR}^{kr} = e^{-\frac{i}{\hbar}E_R^r T}\langle\phi_R^r|e^{-\frac{i}{\hbar}H_k T}|\phi_K^k\rangle \ . \tag{14}$$

The inner product in this equation can be expressed as,

$$\langle\phi_R^r|e^{-\frac{i}{\hbar}H_k T}|\phi_K^k\rangle = \frac{1}{\pi}B_K^k B_R^r(\mathcal{I}_- + \mathcal{I}_+) \ , \tag{15}$$

where

$$\mathcal{I}_- = \int_0^{2\pi}\mathcal{A}(y_2)\mathcal{B}_-(y_2)dy_2 \ , \tag{16}$$

$$\mathcal{I}_+ = \int_{-2\pi}^{0}\mathcal{A}(y_2)\mathcal{B}_+(y_2)dy_2 \ , \tag{17}$$

$$\mathcal{A}(y_2) = \int_0^{4\pi}\mathcal{F}(y_1,y_2)e^{i(K-R)y_1}dy_1 \ , \tag{18}$$

$$\mathcal{B}_\pm(y_2) = \cos[ky_2 \pm (k+K)\pi]\cos[ry_2 \pm (r+R)\pi] \ , \tag{19}$$

$$\mathcal{F}(y_1,y_2) = \exp(-ig\cos y_1/2\cos y_2/2) \ , \tag{20}$$

and $g = 2\xi T/\hbar$. Since $2(K-R)$ is an integer number, $A(y_2)$ can be expressed as the Bessel function of the first kind

$$\mathcal{A}(y_2) = 4\pi i^{2(K-R)}J_{2(K-R)}\left(-g\cos\frac{y_2}{2}\right) \ . \tag{21}$$

This function is an even function of y_2, there is $\mathcal{I}_- = \mathcal{I}_+$. The matrix element now can be written as,

$$U_{KR}^{kr} = 4i^{2(K-R)}e^{-\frac{i}{\hbar}E_R^r T}B_K^k B_R^r(\mathcal{G}_+ + \mathcal{G}_-) \ , \tag{22}$$

where

$$\mathcal{G}_\pm = \int_0^{2\pi}\mathcal{J}(y_2)\cos[\mathcal{K}_\pm(y_2)]\,dy_2 \ , \tag{23}$$

$$\mathcal{J}(y_2) = J_{2(K-R)}\left(-g\cos\frac{y_2}{2}\right) \ , \tag{24}$$

$$\mathcal{K}_\pm(y_2) = (k \pm r)y_2 - (k \pm r + K \pm R)\pi \ . \tag{25}$$

Applying repeatedly integration by parts $\mathcal{G}_\pm$ can be written as

$$\mathcal{G}_\pm = \mathcal{C}_1^\pm + \mathcal{C}_2^\pm + \cdots + \mathcal{C}_n^\pm + \mathcal{D}_n^\pm \ , \tag{26}$$

where

$$\begin{aligned}
\mathcal{C}_{2i-1}^\pm &= (-1)^{i+1} \frac{\mathcal{J}^{(2i-2)}(y_2)\sin\left[\mathcal{K}_\pm(y_2)\right]}{(k\pm r)^{2i-1}}\Bigg|_0^{2\pi} \ , \\
\mathcal{C}_{2i}^\pm &= (-1)^{i+1} \frac{\mathcal{J}^{(2i-1)}(y_2)\cos\left[\mathcal{K}_\pm(y_2)\right]}{(k\pm r)^{2i}}\Bigg|_0^{2\pi} \ , \\
\mathcal{D}_{2i-1}^\pm &= (-1)^{i} \frac{\int_0^{2\pi} \mathcal{J}^{(2i-1)}(y_2)\sin\left[\mathcal{K}_\pm(y_2)\right]dy_2}{(k\pm r)^{2i-1}} \ , \\
\mathcal{D}_{2i}^\pm &= (-1)^{i} \frac{\int_0^{2\pi} \mathcal{J}^{(2i)}(y_2)\cos\left[\mathcal{K}_\pm(y_2)\right]dy_2}{(k\pm r)^{2i}} \ ,
\end{aligned}$$

and $\mathcal{J}^{(n)}(y_2) = \partial^n \mathcal{J}(y_2)/\partial y_2^n$, $n = 1, 2, 3, \ldots$

$\mathcal{J}(y_2)$ is a 4π period function and is an even function: $\mathcal{J}(y_2) = \mathcal{J}(-y_2)$. Consequently $\mathcal{J}^{(n)}(y_2) = (-1)^n \mathcal{J}^{(n)}(-y_2)$ and $\mathcal{J}^{(n)}(0) = 0$ for odd n (taking the limit $y_2 \to 0$). Due to 4π-periodicity, we can take $y_2 = 2\pi - \varepsilon \to 2\pi$, then $\mathcal{J}^{(n)}(2\pi - \varepsilon) = (-1)^n \mathcal{J}^{(n)}(-2\pi + \varepsilon) = (-1)^n \mathcal{J}^{(n)}(2\pi + \varepsilon)$. This implies that $\mathcal{J}^{(n)}(2\pi) = 0$ for odd n. Using these properties of $\mathcal{J}$, we find that

$$\mathcal{C}_{2i}^\pm = 0 \ . \tag{27}$$

The relative momentum k is a solution of Eq. (13) and can be decomposed as $k = N_k + \delta_{N_k}$, where $N_k = 0, 1, 2, \ldots$ for integer K and $N_k = 1/2, 3/2, 5/2, \cdots$ for half integer K. Then Eq. (13) simplifies to

$$\tan(\delta_{N_k}\pi)(N_k + \delta_{N_k}) = \frac{1}{2A_\lambda} \tag{28}$$

First, we note that δ_{N_k} is small for large N_k. We have $N_k \pi \delta_{N_k} \sim 1/2A_\lambda$ and $\delta_{N_k} \sim 1/2\pi N_k A_\lambda$. Therefore for large enough N_k, k behave as

$$k \sim N_k + \frac{1}{2\pi N_k A_\lambda}. \tag{29}$$

Consequently $\delta_{N_k} \neq 0$ and $(k \pm r)$ are non-integer, assuring $\mathcal{C}_{2i-1}^\pm \neq 0$.

This allows us to write $\mathcal{G}_\pm$ as

$$\mathcal{G}_\pm = \mathcal{C}_1^\pm + \mathcal{C}_3^\pm + \mathcal{C}_5^\pm + \cdots + \mathcal{C}_{2n-1}^\pm + \mathcal{D}_{2n}^\pm \ . \tag{30}$$

Let's consider the case of integer K and R. From the definition of $\mathcal{C}_{2i-1}^\pm$, we find,

$$\begin{aligned}
(-1)^{K-R}\left(\mathcal{C}_1^+ + \mathcal{C}_1^-\right) &= \mathcal{M}_0 \left[\frac{\sin(k+r)\pi}{k+r} + \frac{\sin(k-r)\pi}{k-r}\right] \\
&= \frac{\mathcal{M}_0 \cos r\pi \cos k\pi}{k^2 - r^2}\left(2k\tan k\pi - 2r\tan r\pi\right) \ ,
\end{aligned} \tag{31}$$

where $\mathcal{M}_0 = 2J_{2(K-R)}(g)$. From (13) we find that $2k\tan k\pi = 2r\tan r\pi = 1/A_\lambda$. Then $\mathcal{C}_1^+ + \mathcal{C}_1^- = 0$ and $\mathcal{G}_\pm$ reads

$$\mathcal{G}_\pm = \mathcal{C}_3^\pm + \mathcal{D}_4^\pm \ . \tag{32}$$

In the limit $k \gg r$ we can simplify $(k \pm r) \sim k$ in $\mathcal{C}_3^\pm$ and $\mathcal{D}_4^\pm$. We find

$$\mathcal{C}_3^+ + \mathcal{C}_3^- \sim \frac{(-1)^{K+R+1} 2\sin k\pi \cos r\pi}{k^3}\mathcal{M}_2 \ , \tag{33}$$

$$\mathcal{D}_4^+ + \mathcal{D}_4^- \sim \frac{(-1)^{K+R}}{k^4}\mathcal{I}_4 \ , \tag{34}$$

$$\mathcal{I}_4 = 2\int_0^{2\pi} \mathcal{J}^{(4)}(y_2)\cos(ky_2 - k\pi)\cos(ry_2 - r\pi)dy_2 \ , \tag{35}$$

$$\mathcal{M}_2 = -\frac{g}{2} J'_{2(K-R)}(g) \ . \tag{36}$$

Now $\mathcal{G}_+ + \mathcal{G}_-$ becomes

$$\mathcal{G}_+ + \mathcal{G}_- = \frac{(-1)^{K+R+1}}{k^4}\left[2\mathcal{M}_2 k \sin k\pi \cos r\pi - \mathcal{I}_4\right] \ . \tag{37}$$

Since k and r are solutions of Eq. (13), we have

$$\sin k\pi = \frac{(-1)^K}{\sqrt{1+(2kA_\lambda)^2}} \ , \tag{38}$$

$$\cos r\pi = \frac{(-1)^R 2rA_\lambda}{\sqrt{1+(2rA_\lambda)^2}} \ . \tag{39}$$

For large k, $\mathcal{I}_4$ decays to zero due to rapid oscillations of the integrated function in (35), while $2\mathcal{M}_2 k \sin k\pi \cos r\pi$ approaches a constant value. Therefore to leading order we can neglect the correction $\mathcal{I}_4$. Combining with Eq. (38), Eq. (39) and $B_K^k \sim 1/\sqrt{8\pi}$ (see Eq. (12) for the definition of B_K^k) for large k, we find

$$|U_{KR}^{kr}| \sim 2\sqrt{\frac{2}{\pi}} B_R^r \frac{|\mathcal{M}_2| f_{kr}}{k^3} \ , \tag{40}$$

where

$$f_{kr} = \frac{2rA_\lambda}{\sqrt{\left[1+(2kA_\lambda)^2\right]\left[1+(2rA_\lambda)^2\right]}} \ . \tag{41}$$

For a fixed A_λ and large enough k, such that $(2kA_\lambda)^2 \gg 1$, we find

$$|U_{KR}^{kr}| \sim \sqrt{\frac{2}{\pi}} B_R^r \frac{|\mathcal{M}_2|}{k^4} \frac{2r}{\sqrt{1+(2rA_\lambda)^2}} \ . \tag{42}$$

For large r, such that $(2rA_\lambda)^2 \gg 1$ is also valid, and $B_R^r \sim 1/\sqrt{8\pi}$, we find the asymptotic form of $|U_{KR}^{kr}|$

$$|U_{KR}^{kr}| \sim \frac{|\mathcal{M}_2|}{2\pi k^4 A_\lambda} \ . \tag{43}$$

This equation tell us that the matrix element quickly decay along K direction, which is controlled by first order derivative of the Bessel function, and decay as the power law along the k direction, the power exponent is given as -4.

The other cases, such as half-integer K and R, is treated in a very similar way resulting in the same formula (43).
